# Maternal Voluntary Exercise during Pregnancy Enhances the Spatial Learning Acquisition but not the Retention of Memory in Rat Pups via a TrkB-mediated Mechanism: The Role of Hippocampal BDNF Expression

**Published:** 2013-09

**Authors:** Maziar M. Akhavan, Hossein Miladi-Gorji, Mitra Emami-Abarghoie, Manouchehr Safari, Bizhan Sadighi-Moghaddam, Abbas A. Vafaei, Ali Rashidy-Pour

**Affiliations:** 1 Skin Research Center, Laboratory of Protein and Enzyme, Shahid Beheshti University (M.C), Shohada-e Tajrish Hospital, Shahrdari St, Tehran, Iran; 2 Laboratory of Learning and Memory, Research Center and Department of Physiology, School of Medicine, Semnan University of Medical Sciences, Semnan, Iran; 3 Department of Pharmacology, School of Medicine, Semnan University of Medical Sciences, Semnan, Iran; 4 Department of Anatomy, School of Medicine, Semnan University of Medical Sciences, Semnan, Iran; 5 Department of Immunology, School of Medicine, Semnan University of Medical Sciences, Semnan, Iran

**Keywords:** BDNF, Hippocampus, K252a, Memory, TrkB, Offspring, Voluntary exercise

## Abstract

***Objective(s):*** The effect of maternal voluntary exercise on hippocampal BDNF level in rat offspring was studied. In addition, the possible role of hippocampal BDNF receptors in maternal exercise induced enhancement of learning in the rat pups was investigated.

***Materials and Methods:*** Pregnant rats have been randomly assigned to sedentary control or voluntary exercise groups. Each of the exercising pregnant rats was given access to a cage that was equipped with a running wheel until the end of their pregnancy. On post natal day (PND) 36, two groups consisted of 7 male rat pups in each group from sedentary or exercised mothers were sacrificed and the hippocampus was dissected for BDNF proteins level determination. Also, bilateral injection of K252a to the hippocampus was used to block the hippocampal BDNF action on PND59 in the rat pups.

***Results:*** Voluntary exercise during pregnancy significantly increased the level of BDNF protein in the hippocampus of the rat pups on PND36 compared to the control group (*P*=0.048). Inhibiting BDNF action abolished the exercise-induced improvement of learning acquisition in offspring in training trials (*P*=0.0001). No difference was observed in the platform location latency and the time spent in the target in the probe test between two groups.

***Conclusion***: This study demonstrates that voluntary exercise during pregnancy via a TrkB-mediated mechanism enhances the spatial learning acquisition, however, not the retention of memory in the rat pups.

## Introduction

It is well known that physical activity enhances cognitive functions (1), and promotes neurogenesis in the adult mouse dentate gyrus (2). It has been reported that maternal exercise during pregnancy increases the angiogenesis, neurogenesis, learning and memory in rat pups (3-7). Moreover, it has been shown that exercise during pregnancy stimulates the postnatal hippocampal development in offspring (8). It is probable that the connection between the fetus and the exercising muscles in mothers is through the placental barrier, although the mediating factors which contribute to this phenomenon still need to be identified (4-5). Nevertheless, while such a connection is cut on birth, effects of exercise during pregnancy on offspring spatial learning remain at least until the rat pups are weaned (4-7). The molecular events which occupy an important role in mediating the effects of maternal exercise on fetus are still poorly understood. It has been previously shown that serotonergic and noradrenergic systems of the rat pups specifically mediates the enhancing effect of maternal exercise on pup’s spatial learning (4). On the other hand, several reports suggest that brain derived neurotrophic factor (BDNF) is an important modulator of physical activity’s effects on neural functions such as synaptic plasticity, neurogenesis, learning and memory (9-12). 

It is well documented that physical exercise can elevate BDNF level in hippocampus (13-15) which acts via TrkB receptors and blocking BDNF signaling prevents the enhancement in cognitive functions following exercise (15). However, there are controversial reports regarding the effect of maternal treadmill running in pregnancy on hippocampal BDNF mRNA about the time that the rat pups are weaned. Parnpiansil *et al *(2003) reported a significant decrease and Kim *et al* (2007) reported a significant increase in the hippocampal BDNF mRNA in rat pups born from exercised mothers compared with the control group at PND 28 and PND 29, respectively (5,7). Although animal exercise models which consisted of forced procedures enable the experimenter to control the intensity of the exercise, but it may increase the stress of the pregnant animal and this should be considered in evaluation of the observed effects (4). Voluntary exercise is free of such a stress and may provide us with a better method to evaluate the effects of maternal exercise on offspring. These suggestions lead to the hypothesis that maternal voluntary exercise during pregnancy may increase the expression of hippocampal BDNF in offspring. In addition, the role of hippocampal BDNF in mediating the enhancing effect of maternal voluntary exercise on learning acquisition in rat pups was investigated.

## Materials and Methods

***Animals and experimental groups***

**Figure 1:**
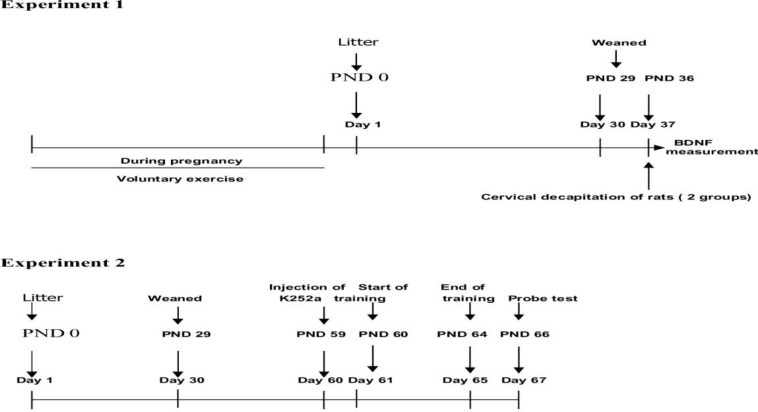
Timelines of experiments

Male Wistar rats (21010 g) were allowed to mate with female virgin wistar rats (21010 g) during a 24 hr period. Female rats were checked for the presence of a vaginal plug twice at midnight and at 5 am the next day. Pregnant rats have been randomly assigned to the sedentary control (S. control) or voluntary exercise (V. exercise) groups (N=20 in each group) and were housed individually in cages with a 12 hr light/dark cycle at 22-24 C temperature, with food and water *ad libitum*. All experimental procedures were carried out in accordance with the national institute of health guide for the care and use of laboratory animals. Also care was taken to use the minimum number of animals and to minimize their suffering. From day 21 after mating, pregnant rats were checked twice daily for birth, at 9 am and 6 pm. The day that the rat pups were first observed was taken as PND 0. On post natal day (PND) 36 (One week after weaning to avoid the stress of weaning), two groups consisted of 7 male rat pups in each group from sedentary or exercised mothers (one pup from each colony) were sacrificed and the brains were dissected. The hippocampus was dissected and kept at -70C until used for BDNF proteins level determination. On PND 59 (according to Paxinos and Watson atlas, two-month-old rat hippocampus was used to inject), 28 male rat pups were used for water maze (WM) test designed to study the effects of blockade of BDNF receptor (TrkB) on the exercise-induced improvement of learning. In this experiment, male rat pups born from sedentary or exercised mothers were randomly assigned to four groups (n=7 in each group): sedentary group that received cytochrome C (Cyt C) (Sed/Cyt C), sedentary group that received K252a (Sed/K252a), exercised group that received cytochrome C (Exc/Cyt C), exercised group that received K252a (Exc/K252a) (see Figure 1. timelines of experiments).

***Voluntary exercise paradigm***

Each of the exercising pregnant rats was given all day/night access to a cage that was equipped with a running wheel (diameter = 34.5 cm, width = 9.5 cm) (NovidanTeb, Iran) that was freely rotated against a resistance of 100 g, until the end of their pregnancy period. Each wheel was attached to a counter that monitored its revolutions (which were recorded daily at 6 am). After the delivery, each mother along with pups in this group was transferred to a normal cage without having an access to a running wheel. Pregnant rats in the sedentary group were placed individually in the same normal cages without access to a running wheel (3-4).

***Surgical procedures and*** ***TrkB receptors blocking protocol***

We administered a Trk receptor inhibitor (K252a) or cytochrome C (Cyt C), (both from Sigma, St. Louis, MO, USA) via injection of fluorescent latex microspheres directly into the right and left hippocampus (Lumafluor Corp. 1213 Silver strand drive, Naples, FL 34110 USA). Intrahipocampal injection of K252a has been shown to effectively block the action of BDNF in vivo (15, 16). 

Successful delivery of bioactive agonists or antagonists into highly localized brain regions such as hippocampus using latex microbeads has been reported (13, 15, 17-19). This is an effective and simple method which in several reports replaced more invasive injection techniques such as cannula injection or micro-pump implantation. Several previous studies have also reported the effective release of bioactive agents by microbeads for 5 days (13, 17, 19). With the application of microbeads, the microbead latex carrier is remained in the injection site and the transported bioactive agent is released to disperse to other areas of the target region (15).

The microspheres preparations for injections have been described elsewhere (13, 17, 19-20). The bioactive agents were passively absorbed to the microspheres by incubating overnight at 4C with a 1:5 mixture of microspheres to K252a (46.8 ng/ l sterile water) (20) or Cyt C (100 ng/l sterile water) (16, 18, 21). Cyt C was used, as the protein drug to the control as it has been successfully employed as a standard control for micro bead injections (13, 17, 20). During the next day, the solution was centrifuged at 14000g for 30 min and the microbeads were resuspended in sterile water at a 10% concentration. 

The injection of neurotrophins using microspheres as a vehicle has provided neurotrophic activity comparable to that of free neurotrophin, and has been shown to retain activity for at least 4-5 days (13, 19). Here we use this capability of microspheres to deliver antagonists for a period of 5 days during acquisition trial. Therefore, all animals (N=7 male rat pups /in each group) received the injection once, one day prior to their WM test, such that a sufficient recovery time permitted all animals to begin WM test the next day (see Figure 1. timelines of experiments 2). All rats were anesthetized (IP), with a combination of 70 mg/kg of ketamine hydrochloride and 10 mg/kg of xylazine (Purchased from animal pharmacy), once before receiving the drug injection. For injection into the hippocampus the animals head was secured in a stereotaxic frame, shaved and bathed with betadine. A midline incision of the scalp was made. The skull was cleaned and dried by cotton swabs. Microspheres were injected into the right and left hippocampus (3.8 mm from Bregma, 2.6 mm from the midline and 3.7 mm vertically) with either 2 l of K252a or Cyt C as described above over a period of 15 min (15, 18-20). 

***Testing learning and memory using the water maze***

A detailed description of the apparatus and the video tracking system has been given in our previous reports (17, 22). The water maze was a black circular pool (140 cm in diameter and 60 cm height) that was filled up to a 25 cm depth with 22 2°C water temperature (4, 20, 22). 

**Figure 2:**
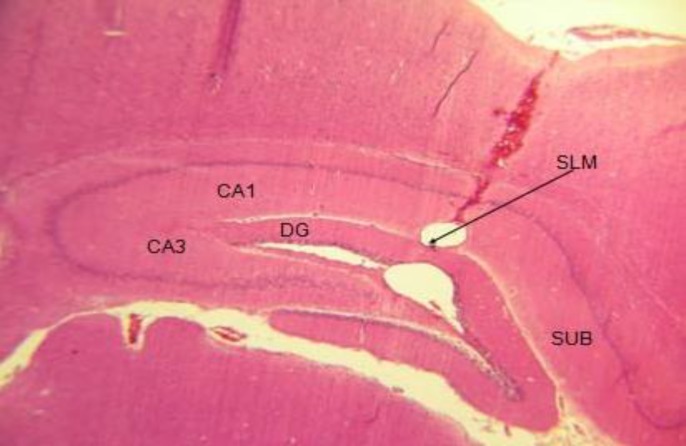
Tissue section in the coronal plane of the hippocampus showing the injection site of the microbeads  into the stratum lacunosum moleculare (SLM). For convenience, the hippocampal areas have been labeled

**Figure 3:**
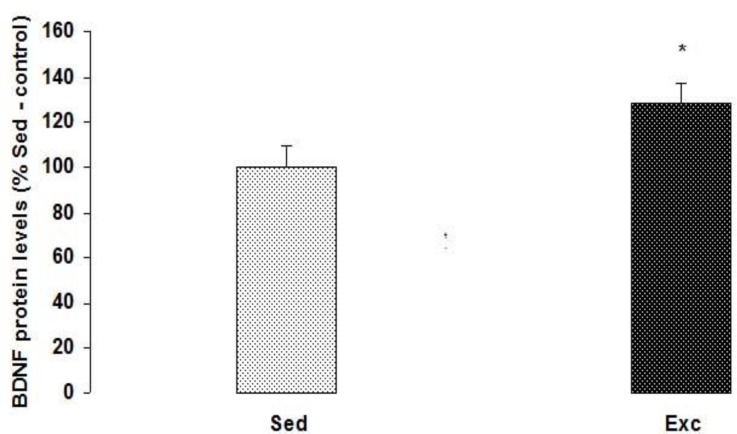
The effect of maternal voluntary exercise during pregnancy on BDNF protein level in the hippocampus. The BDNF levels are displayed as the percentages of the Sed-control levels (represented by the 100% line). The data are expressed as the mean ± SEM. Maternal voluntary exercise during pregnancy significantly increased the levels of BDNF in the hippocampus of the rat pups.*represent the significant difference between two groups (*P*=0.048

**Figure 4:**
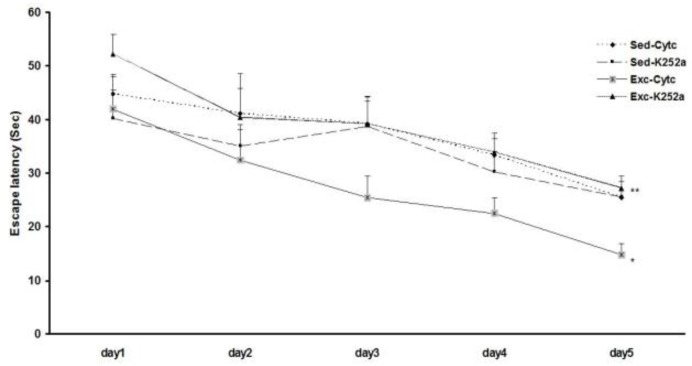
The effect of blocking BDNF action on pups learning as measured by the WM task. Exercise effectively improved learning, as evidenced by the fact that Exc/Cyt C group took significantly less time to find the platform on day 5 of training. The blockade of BDNF action during exercise abolished the exercise-induced enhancement of learning because the exercising rats that were given K252a injection took longer time to find the platform compared to the exercising controls (Exc/K252a and the Exc/Cyt C). *represents the significant difference between the Exc/Cyt C and Sed/Cyt C groups, and **represents the significant difference between the Exc/K252a and Exc/Cyt C groups

**Figure 5:**
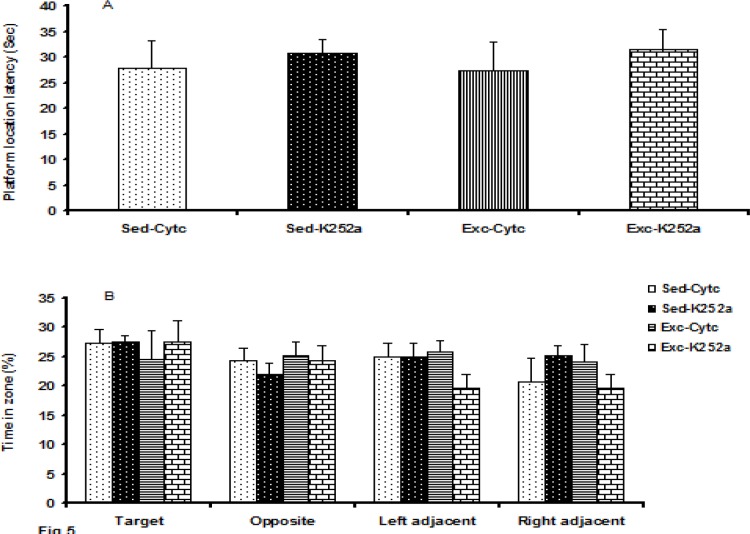
The effect of maternal voluntary exercise during pregnancy on pup’s memory retention as measured by the WM task using the probe trial. (A) The mean latency to reach the previous location of the platform. (B) The mean time spent in within a target zone or on an equivalent location in other quadrants, was expressed as a percentage of the total time spent in both of the zones. (A) The platform location latency and (B) percentage of the time spent in each equivalent location in other quadrants did not differ significantly between groups (*P*> 0.05)

The WM protocol was a stringent protocol consisting of two trials per day for 5 consecutive days, which has been shown to be a good discriminative test for studying the effects of exercise on learning and memory (13, 19). During each trial, the rat was placed into the water facing the wall from one of the four cardinal points of the compass (N, E, S, and W), which varied from each trial to trial in a random order. The rats were guided by the hand to the platform if they failed to locate it within 60 sec. The rat was allowed to stay on the platform for 20 sec during the inter-trial interval. After the last trial, the animal was towel dried and returned to its home cage with no access to a running wheel. The escape latency (platform search time) for each trial was recorded.

A spatial probe test was performed 2 days after the last acquisition trial, during which the platform was removed. The rats were allowed to swim for 60 sec, and the latency to reach the previous location of the platform and the time spent swimming in each quadrant (target, opposite, left and right adjacent quadrants) were recorded. The velocity of each animal was also calculated. Swim paths were semi-automatically recorded by a video tracking system reports (20, 22).

***Protein measurements***

The hippocampal extracts were prepared in lysis buffer (137 mM NaCl, 20 mM Tris–HCl pH 8.0, 1% NP-40, 10% glycerol, 1 mM phenylmethyl sulfonyl fluoride (PMSF), 10 μg/mL aprotinin, 1 μg/mL leupeptin, and 0.5 mM sodium orthovanadate). The homogenates were centrifuged to remove insoluble materials (12500 g for 20 min at 4^°^C), and the total protein concentration was determined according to the Micro BCA procedure (Pierce, Rockford, IL, USA). The BDNF protein levels were assessed using an E-Max ELISA kit (Promega, WI, USA), according to the manufacturer’s recommendations which has been described elsewhere (13, 20, 23).

***Statistical analysis***

The data expressed as the mean standard error of the mean (SEM). A three-way repeated measures ANOVA (days) followed by a Tukey’s test was conducted for data between multiple groups in WM test. In addition, a student t-test was used when two groups were comparing. The statistical differences were considered to be significant at *P*0.05.

## Results

The injection sites of the microbeads in the hippocampus were confirmed using tissue microscopy using a light microscope with a 40× objective lens. Figure 2 represents the tissue section in the coronal plane that contained the site of the injection in the hippocampus using *cresyl fast violet* (Nissl) staining method. The area (stratum lacunosum molecular) of the microbead concentration is consistent with the previous studies that used the same method for the local delivery into the hippocampus of rats (13, 17, 19). 

***BDNF protein*** ***levels***

Here we report that voluntary exercise during pregnancy significantly (t_12_=-0.2, *P=*0.048) increased the expression of BDNF protein in the hippocampus of the rat pups on PND 36 compared to the control group (Figure 3). 

***Hippocampal TrkB receptors blocking abolished the effects of maternal exercise on pups learning***

The acquisition data during the 5 days of training in the WM are illustrated in Figure 4. Three-way repeated measures ANOVA (K252a treatment × exercise× training days) were used to analyze the escape latencies during training. All groups learned to locate the platform during the 5 successive days of training as indicated by decreasing escape latencies as training progressed (F_4, 120_=14.6, *P=*0.0001). The effect of exercise was statistically significant (F_1,120_=4.54, *P=*0.012): the Exc/Cyt C group exhibited significantly shorter escape latencies only on day 5 of the WM training than those of the Sed/Cyt C control groups (*P=*0.026). While, the Exc/K252a group exhibited significantly longer escape latencies only on day 5 of the WM training than those of the Exc/Cyt C control groups (*P*=0.008). There was a significant interaction between K252a treatment and exercise (F_1, 120_=16.93, *P=*0.0001), however, no significant interactions were noted between K252a treatment and exercise and days (F_12, 120_=0.42, *P=*0.99). These findings indicate that a blockade of BDNF action reverses the maternal exercise-induced enhancement of learning, but, does not have any effect on sedentary rats.

Data related to the distance swam to reach the platform followed a similar pattern to the latency. All groups traveled shorter distances to reach the platform as training progressed (F4, 120=15.6, *P*=0.002). Exc/Cyt C group exhibited significantly shorter distances only on day 5 of the WM training than those of the Sed/Cyt C control groups (*P*=0.002). While, the Exc/K252a group exhibited significantly longer distances only on day 5 of the WM training than those of the Exc/Cyt C control groups (*P*=0.0001) (data not shown). 

***Hippocampal TrkB receptor blocking was no effect on memory retention in pups of exercised mothers using the probe trial***

The data for the memory retention test are shown in Figure 5. No difference was found in the platform location latency (Figure 5A) (F 3, 27 =0.178, *P*=0.91) and the time spent (Figure 5B) in the target (F3, 27 =0.21, *P*=0.89) or opposite zone (F3, 27 =0.34, *P*=0.79) in the probe test between groups. As a control for differences in WM performance, we also recorded each animal’s swimming speed. No difference was found in the swimming speeds for any of the four groups (F3, 27 =1.20, *P*=0.33).

## Discussion

Voluntary exercise during pregnancy significantly increased the level of BDNF protein in the hippocampus of the rat pups on PND 36 compared to the control group. 

The present result is in accordance with Lee *et al* (2006) and Kim *et al* (2007), reporting an increase in BDNF mRNA level in rat pups from forced exercised mothers (5, 6). 

On the other hand, Parnpiansil *et al* (2003) reported a significant decrease in the hippocampal BDNF mRNA in rat pups born from forced exercised mothers compared to the control group at PND 28 (7). However, it is important to consider that the forced exercise models consisted of stressful experiences that increase the corticosterone level in mother blood and therefore submitting the pregnant rats to such a model may affect the results on offspring through the elevated blood corticosterone level (4). In addition, it seems that the stress level is an important factor which may contribute to the observed effects on progeny (24). The possible effect of prenatal exposure to stress on BDNF expression in offspring is further supported by reports which suggest that prenatal exposure to the stress may decrease the expression of BDNF in different areas of the adult rat brain (25). Another factor which should be considered is that the voluntary exercise is usually performed by the rats during the dark period of the light/dark cycle (which is their active period) but the forced exercise models are usually performed during the light period. It has been suggested that the influence of exercise on cell proliferation and neurogenesis is modulated by both circadian phase in which the exercise is performing and the amount of daily exercise (26). 

In this study, we have blocked the action of BDNF on TrkB receptors using K252a, which is a selective antagonist for Trk receptor family (27). TrkB receptors blocking abolished the effects of maternal exercise on pups learning during the 5 days of training in the WM. The WM protocol is a stringent protocol consisting of two trials per day for 5 days, which has been suggested to be a good discriminative protocol for comparing the effects of exercise on learning and memory (4, 13, 19). It has been reported that K252a block the BDNF-evoked excitatory potentials in hippocampal neurons (16). In addition, it has been shown that injection of K252a directly to the hippocampus fully abolished the exercise induced increase of BDNF and TrkB receptors mRNA (15). Our current results confirm that maternal voluntary exercise enhances the learning acquisition in the rat pups via BDNF receptor.

Consequently, there is a question that why TrkB receptor blocking could specifically suppress the enhancing effect of maternal voluntary exercise on pups learning. One possible explanation is that maternal exercise during pregnancy may increase the number of the TrkB receptors in the rat pups hippocampus. This theory is in agreement with the some reports indicating that exercise up-regulates the TrkB receptors in brain or spinal cord (15, 28). 

Previously, the importance of BDNF as a mediator of the effects of exercise on cognitive function has been demonstrated (15). Activation of the TrkB receptors by BDNF promotes the phosphorylation of synapsin I which in turn results in neurotransmitter release. That is end up to synaptic plasticity, neurogenesis, and learning and memory (7, 10-12).

In line with our previous study (4), we revealed that maternal voluntary wheel running selectively enhanced the acquisition phase, while no significant changes were detected in the retention phase in rat pups whose mothers have been submitted to voluntary wheel running in compared with swimming exercise. Also, in the present study, application of the BDNF inhibitor during the voluntary exercise was sufficient to blunt the exercise-induced improvement of learning, while spatial memory was not affected. There are some other factors that may be of importance in part of the differences observed between the different exercise models. This explanation is in accordance with the findings of others indicating that short lasting or mild prenatal stress could enhance neuronal differentiation in the hippocampus and spatial learning in offspring (4, 24) and minimizes the alterations of the utero-placental blood flow (4, 6). This might be an important factor in mediating the exercise effects on fetus. This question cannot be answered by the present data, and requires a different experimental protocol.

We found no difference in the swimming speeds for all four groups. Thus, physical activity was not significantly altered by TrkB receptor blocking.

## Conclusion

This study demonstrates a central role for BDNF signaling through TrkB receptors activation in mediating the maternal voluntary exercise induced effects on offspring. Blocking the TrkB receptors abrogated the effect of maternal V.exercise during pregnancy on offspring learning acquisition at PND 59 which shows that prenatal exposure to maternal V.exercise produce long term effects on progeny, although it may be less prominent in older ages. 

## References

[1] Coles K, Tomporowski PD (2008). Effects of acute exercise on executive processing, short-term and long-term memory. J Sport Sci.

[2] Van Praag H, Kempermann G, Gage FH (1999). Running increases cell proliferation and neurogenesis in the adult mouse dentate gyrus. Nat Neurosci.

[3] Akhavan MM, Foroutan T, Safari M, Sadighi-Moghaddam B, Emami-Abarghoie M, Rashidy-Pour A (2012). Prenatal exposure to maternal voluntary exercise during pregnancy provides protection against mild chronic postnatal hypoxia in rat offspring. Pak J Pharm Sci.

[4] Akhavan MM, Emami-Abarghoie M, Safari M, Sadighi-Moghaddam B, Vafaei AA, Bandegi, AR (2008). Serotonergic and noradrenergic lesion suppress the enhancing effect of maternal exercise during pregnancy on learning and memory in rat pups. Neuroscience.

[5] Kim H, Lee SH, Kim SS, Yoo JH, Kim CJ (2007). The influence of maternal treadmill running during pregnancy on short-term memory and hippocampal cell survival in rat pups. Int J Dev Neurosci.

[6] Lee HH, Kim H, Lee JW, Kim YS, Yang HY, Chang HK (2006). Maternal swimming during pregnancy enhances short-term memory and neurogenesis in the hippocampus of rat pups. Brain Dev.

[7] Parnpiansil P, Jutapakdeegul N, Chentanez T, Kotchabhakdi N (2003). Exercise during pregnancy increases hippocampal brain-derived neurotrophic factor mRNA expression and spatial learning in neonatal rat pup. Neurosci Lett.

[8] Bick-Sander A, Steiner B, Wolf SA, Babu H, Kempermann G (2006). Running in pregnancy transiently increases postnatal hippocampal neurogenesis in the offspring. Proc Natl Acad Sci USA.

[9] O’Callaghan RM, Ohle R, Kelly AM (2007). The effects of forced exercise on hippocampal plasticity in the rat: A comparison of LTP, spatial- and non-spatial learning. Behav Brain Res.

[10] Stranahan AM, Zhou Y, Martin B, Maudsley S (2009). Pharmacomimetics of exercise: Novel approaches for hippocampally-targeted neuroprotective agents. Curr Med Chem.

[11] van Praag H, Shubert T, Zhao C, Gage FH (2005). Exercise enhances learning and hippocampal neurogenesis in aged mice. J Neurosci.

[12] Van Praag H (2008). Neurogenesis and exercise: Past and future directions. Neuromolecular Med.

[13] Ding Q, Vaynman S, Akhavan MM, Ying Z, Gomez – Pinilla F (2006). Insulin- like growth factor 1 interfaces with brain -derived neurotrophic factor - mediated synaptic plasticity to modulate aspects of exercise - induced cognitive function. Neuroscience.

[14] Neeper SA, Gomez-pinilla F, Choi J, Cotman CW (1996). Physical activity increases mRNA for brain-derived neurotrophic factor and nerve growth factor in rat brain. Brain Res.

[15] Vaynman S, Ying Z, Gomez-Pinilla F (2003). Interplay between BDNF and signal transduction modulators in the regulation of the effects of exercise on synaptic-plasticity. Neuroscience.

[16] Kafitz KW, Rose CR, Thoenen H, Konnerth A (1999). Neurotrophin-evoked rapid excitation through TrkB receptors. Nature.

[17] Akhavan MM, Emami-Abarghoie M, Sadighi-Moghaddam B, Safari M, Yousefi Y, Rashidy-Pour A (2008). Hippocampal angiotensin II receptors play an important role in mediating the effect of voluntary exercise on learning and memory in rat. Brain Res.

[18] Lom B, Cohen-Cory S (1999). Brain-derived neurotrophic factor differentially regulates retinal ganglion cell dendritic and axonal arborization in vivo. J Neurosci.

[19] Vaynman S, Ying Z, Gomez-Pinilla F (2004). Hippocampal BDNF mediates the efficacy of exercise on synaptic plasticity and cognition. Eur J Neurosci.

[20] Miladi-Gorji H, Rashidy-Pour A, Fathollahi Y, Akhavan MM, Semnanian S, Safari M (2011). Voluntary exercise ameliorates cognitive deficits in morphine dependent rats: The role of hippocampal brain-derived neurotrophic factor. Neurobiol learn mem.

[21] Vaynman S, Ying Z, Gómez-Pinilla F (2004). Exercise induces BDNF and synapsin I to specific hippocampal subfields. J Neurosci Res.

[22] Ebrahimi S, Rashidy-Pour A, Vafaei AA, Akhavan MM (2010). Central β-adrenergic receptors play an important role in the enhancing effect of voluntary exercise on learning and memory in rat. Behav Brain Res.

[23] Adlard PA, Cotman CW (2004). Voluntary exercise protects against stress - induced decreases in brain - derived neurotrohic factor protein expression. Neuroscience.

[24] Fujioka A, Fujioka T, Ishida Y, Maekawa T, Nakamura S (2006). Differential effects of prenatal stress on the morphological maturation of hippocampal neurons. Neuroscience.

[25] Van den Hove DL, Lauder JM, Steepens A, Prickaerts J, Blanco CE, Steinbusch HW (2006). Prenatal stress in the rat alters 5-HT1A receptor binding in the ventral hippocampus. Brain Res.

[26] Holmes MM, Galea LA, Mistlberger RE, Kempermann G (2004). Adult hippocampal neurogenesis and voluntary running activity: circadian and dose-dependent effects. J Neurosci Res.

[27] Knüsel B, Hefti F (1992). K-252 compounds: modulators of neurotrophin signal transduction. J Neurochem.

[28] Skup M, Czarkowska-Bauch J, Dwornik A, Macias M, Sulejczak D, Wiater M (2000). Locomotion induces changes in Trk B receptors in small diameter cells of the spinal cord. Acta Neurobiol Exp (Wars).

